# Interrelation Among Anthropometric Indices, Body Composition, Physical Fitness, and Glycated Hemoglobin in a Cohort of Young Female University Students: A Cross-Sectional Study

**DOI:** 10.7759/cureus.80324

**Published:** 2025-03-10

**Authors:** Safia Fathima Anver Pasha, Salwa Sadathulla Syed, Asmaa Mohamed Saber Mohamed Mohamed Sharafeldin, Elham Hassan Nazari Fard, Suresh Kumar Srinivasamurthy, Manjunatha Goud

**Affiliations:** 1 Internal Medicine, RAK Medical and Health Sciences University (RAKMHSU), Ras Al Khaimah, ARE; 2 Pharmacology, RAK Medical and Health Sciences University (RAKMHSU), Ras Al Khaimah, ARE; 3 Biochemistry, RAK Medical and Health Sciences University (RAKMHSU), Ras Al Khaimah, ARE

**Keywords:** anthropometric indices, body composition, female university students, glycated hemoglobin, overweight, physical fitness, sedentary behaviour

## Abstract

Introduction: Glycated hemoglobin (HbA1c) reflects average blood glucose levels over the preceding three months, independent of fasting state. It is widely used for diagnosing type 2 diabetes and prediabetes and monitoring glucose control in patients with diabetes. This study highlights how anthropometry, body composition, and physical fitness affect glycated hemoglobin (HbA1c) levels in a cohort of female university students.

Materials and methods: This cross-sectional study, conducted between October 2023 and January 2024 among university female students (n=86; ages 17-22 years) in the United Arab Emirates (UAE), aimed to determine the relationship between HbA1c, anthropometric indices, physical fitness, and body composition. Participants were recruited in person, provided written informed consent, and completed demographic and physical activity questionnaires. Weight, body mass index (BMI), visceral fat, and muscle and fat percentage were measured using a body composition monitor scale (Omron HBF-514C, OMRON Healthcare, Inc., Kyoto, Japan). A stadiometer was used to measure height and an electronic sphygmomanometer to measure blood pressure. The waist and hip circumferences were measured using a measuring tape, and the waist-to-hip ratio was further calculated. Glycated hemoglobin (HbA1c) was measured using an immunofluorescence assay analyzer (Getein 1160 Immunofluorescence Quantitative Analyzer, Getein Biotech, Inc., Nanjing, China).

Results and discussion: The main objective of our study was to establish a relationship between anthropometric measurements and body composition with physical fitness levels, including sedentary time, and how it influences HbA1c levels in young female students. Of the 90 participants, data from 86 were analyzed. Significant correlations were found between HbA1c and diastolic blood pressure (BP) (r=0.227) and sedentary hours (r=0.206). Linear regression analysis revealed sedentary behavior (p=0.021) and diastolic BP (p=0.034) as significant predictors of HbA1c levels. In conclusion, our research study provides valuable insights into how lifestyle factors such as sedentary lifestyle and diastolic BP influence glycemic (HbA1c) control in a specific cohort of young female university students of ages 17-22 years. Although BMI was not a significant predictor in our study, sedentary hours and diastolic blood pressure were independently linked to HbA1c levels.

## Introduction

Glycated hemoglobin (HbA1c) offers the average blood glucose levels over the past three months independent of the fasting state of the individual. The HbA1c test has screening, diagnostic, and monitoring purposes, where it’s used to diagnose type 2 diabetes mellitus and prediabetes and monitor the glycemic control in people with diabetes. It can be used in people experiencing classic symptoms of diabetes such as polydipsia, polyuria, polyphagia, fatigue, weight loss with blurry vision, increased recurrent infections, and peripheral paresthesis seen in severe hyperglycemia [1]. It is essential to identify and diagnose individuals with undiagnosed diabetes, as many patients first seek medical care only after developing complications of the disease. Significant risk factors for diabetes in the United Arab Emirates (UAE) and worldwide include age (more than 35 years), family history, hypertension, high body mass index (BMI), and high waist-to-hip ratio [2].

The control of diabetes is lifelong, which brings in the difficulty of managing the disease. It contributes to various microvascular complications such as retinopathy, nephropathy, and neuropathy and macrovascular complications such as myocardial infarction, stroke, and peripheral vascular disease with diabetic foot or ulcers, thus increasing the overall morbidity and mortality [3]. Consequently, the recommended course of action is primary prevention by screening to identify the disease as soon as possible and control it through lifestyle modifications such as physical activity, exercise regimes, and maintaining a ‘normal’ body mass index (BMI) [4]. That begs the question of whether there’s a direct relation between physical activity and body mass index (BMI) with diabetes and, hence, if any relation exists between HbA1c levels and body compositions among adolescents.

The importance of prevention in this age group, i.e., 17-22 years, is to prevent the development of the disease and promote early screening. A large proportion of patients with diabetes don’t visit clinics due to the asymptomatic nature of the disease. Different measures can be taken to preclude type 2 diabetes in younger populations, such as regular exercise, maintaining a healthy weight, and a proper balanced diet [5].

The main objective of our study was to establish a relationship between anthropometric measurements and body composition with physical fitness levels, including sedentary time, and how it influences HbA1c levels in young female students. This study is crucial in identifying factors influencing glycemic control. If left uncontrolled, it leads to a diagnosis of diabetes, a major public health concern associated with severe complications. By examining the relationship between body composition, anthropometric measures, and physical activity levels with HbA1c levels, it aims to inform lifestyle modifications and promote metabolic health. This, in turn, will enhance early detection and help reduce the incidence of diabetes and its complications in the future.

## Materials and methods

The study was conducted between October 2023 and January 2024. All the participants gave written informed consent before their participation. The study was approved by Ras Al Khaimah Medical and Health Sciences University, Research and Ethics Committee (RAKMHSU-REC No:119-2022/23-UG-M). The participants were recruited by personally meeting them and getting an informed consent with the aim of determining a relationship between HbA1c, physical fitness, and body composition. After obtaining informed consent, the participants’ demographic details were recorded, and they were asked to fill out the World Health Organization (WHO) Global Physical Activity Questionnaire (GPAQ) (Appendix 1) [6]. Ninety females aged 17-22 years volunteered to participate in this study. Participants with a self-reported status of anemia and clinically diagnosed diabetes or on treatment specific for diabetes were excluded, according to which the data of four participants were not sent for data analysis. The family history of diabetes and hypertension in our participants was not considered at the time of recruitment.

Anthropometric measurements were taken after filling out the Global Physical Activity Questionnaire (GPAQ). The levels of physical activity include moderate and vigorous intensity activities grouped into three domains: activity at work, travel to and from places, and recreational activities, following which metabolic equivalents of tasks (METs) were calculated. The number of METs assigned to moderate and vigorous intensity activities were four and eight, respectively. Weight, body mass index (BMI), visceral fat, and muscle and fat percentage were measured using the body composition monitor scale (Omron HBF-514C, OMRON Healthcare, Inc., Kyoto, Japan) while being barefoot. The height was obtained using a stadiometer. By using a measuring tape, the waist circumference was measured at the midpoint between the top of the iliac crests and the lower edge of the last perceptible ribs. The widest part of the buttocks was measured for the hip circumference, with the tape parallel to the ground. Blood pressure was measured at rest whilst seated by an electronic sphygmomanometer (Omron M3W Automatic Upper Arm Blood Pressure Monitor, OMRON Healthcare, Inc., Kyoto, Japan) [7].

The body roundness index (BRI) is a newly developed geometrical index that combines height and waist circumference to quantify individual body shape [8]. It aims to predict total body fat and visceral fat more precisely than traditional measurements like BMI. The BRI serves as a useful tool for evaluating health status, offering a detailed understanding of body composition compared to traditional methods. Such models consider the human body shape as an ellipse to relate waist circumference and height in the minor axis and major axis of the ellipse, respectively [8].

BRI was calculated using an online calculator by the following formula:

BRI = $364.2 - 365.5 \times \sqrt{1 - \left(\frac{\text{waist circumference in centimeters}}{2\pi}\right)^2 / \left(0.5 \times \text{height in centimeters}\right)^2}$

HbA1c was measured with a whole blood sample of the participant using the immunofluorescence assay analyzer (Getein 1160 Immunofluorescence Quantitative Analyzer, Getein Biotech, Inc., Nanjing, China). It was expressed in percentage, and all the values of the participants were stored in this analyzer with their identification details.

The immunofluorescence assay analyzer provides a rapid measurement of HbA1c using the dogma of immunofluorescence. The whole blood sample moves over the sample swab strip on the absorbent pad. The HbA1c present in the sample binds to its antibody conjugated with ﬂuorescent microspheres. The concentration of HbA1c is proportional to the intensity of the ﬂuorescence signal [9].

A body composition monitor scale works by the principle of bioelectrical impedance. Bioelectrical impedance analysis (BIA) is centered on the electrical properties of biological tissues. When sending a weak alternating electric current into the body, electricity flows along highly conductive body tissue [10]. By this method, the composition monitor scale measures body fat percentage, BMI, skeletal muscle, visceral fat, and body weight, as current can pass through fat and muscle, hence providing an adequate measurement. The data was analyzed by IBM SPSS Statistics for Windows, Version 29 (Released 2023; IBM Corp., Armonk, New York, United States), descriptive statistics, and the linear regression model, and Pearson correlation was used to present the results. The results were adjusted for confounders such as age, muscle percent, visceral fat, BMI, total METs-mins, total days for all domains, systolic blood pressure (SBP), diastolic blood pressure (DBP), BRI, and sedentary time. A p-value less than 0.05 is considered statistically significant. 

## Results

The study population included 90 female students aged 17-22 years, with data analysis conducted for 86 of them. Four participants were excluded from our study following the exclusion criteria since one participant was a known diabetic on insulin, while the remaining three participants’ HbA1c values were missing or unknown due to technical error. The mean ± SD age of our participants was 19.60 ± 1.40 years. All participants were enrolled either in the medical, pharmacy, or nursing programs of our university. Descriptive statistics of all the participants are illustrated in Table 1.

**Table 1:** Descriptive statistics of the participants *Domains include: Activity at work, travel to and from places, recreational activities, and sedentary behavior.

Descriptive Statistics		
Total number of participants (n=86)	Mean	Standard deviation
Age (years)	19.60	1.408
Weight (kg)	65.90	13.66
Fat percentage (%)	39.44	8.55
Muscle percentage (%)	24.57	3.68
Visceral fat	4.49	1.53
Height (cm)	159.86	6.47
Systolic blood pressure (SBP) (mmHg)	104.36	12.49
Diastolic blood pressure (DBP) (mmHg)	75.52	9.23
Waist-to-hip ratio	0.82	0.05
Skinfold thickness (mm)	23.27	5.56
Glycated hemoglobin (HbA1c) (%)	4.69	0.82
Body mass index (BMI) (kg/m^2^)	25.69	4.44
Body roundness index (BRI)	3.35	1.17
Total metabolic equivalent of task in minutes per week (METs-min/week) for all domains*	2590.70	3448.37
Sedentary time per day (min)	550.23	302.78

The anthropometric measurements taken include the weight, height, skin-fold thickness, waist-to-hip ratio, fat percentage, muscle percentage, and visceral fat. The mean ± SD weight of our participants was 65.90 ± 13.66 kg, and the mean visceral fat was 4.49%.

The mean ± SD HbA1c value was 4.69 ± 0.82% in our participants. This falls into the normal or non-diabetic range (HbA1c <5.7%) as recommended by the American Diabetes Association (ADA) [3]. Participants with clinically diagnosed diabetes and on treatment specific for diabetes were not considered in the study (as part of the exclusion criteria). The participants with HbA1c values in the prediabetic and diabetic ranges (four to five participants, respectively) were informed of their abnormal HbA1c values for further precaution. The overall mean ± SD BMI was 25.69 ± 4.44 kg/m². This value lies in the overweight range as described by the WHO (25-29.9 kg/m²) [11]. Overall, 18 participants (20.9%) were obese, and 31 participants (36%) were overweight.

To evaluate physical fitness or activity in our participants, we narrowed it down to include the total metabolic equivalent of task (METs) in minutes, the total number of days spent in moderate or vigorous physical activity, and the amount of time spent sitting/sedentary time in minutes. The mean ± SD sedentary time spent per day was 550.23 ± 302.78 minutes, or roughly 9.1 hours per day. There was a correlation established between sedentary time per day (in minutes) and HbA1c with an r-value of 0.206 and 95% CI (-0.007-0.4). Systolic blood pressure (SBP) and diastolic blood pressure (DBP) were measured, resulting in a mean ± SD of 104.36 ± 12.49 mmHg and 75.52 ± 9.23 mmHg, respectively. A correlation was established between DBP and HbA1c with an r-value of 0.227 and 95% CI (0.016 - 0.419).

Following linear regression analysis between HbA1c as a dependent variable and SBP, DBP, muscle percentage, sedentary time spent per day, and total METS-mins as independent variables, sedentary time and DBP showed p=0.021 and p=0.034, respectively, with p<0.05 considered statistically significant as shown in Table 2. Hence, sedentary time and DBP were statistically significant predictors of HbA1c in our study. 

**Table 2:** Linear regression between HbA1c and other variables A p-value less than 0.05 is considered statistically significant. MET: metabolic equivalent of task; HbA1c: glycated hemoglobin

Variables	Standardized Coefficients - Beta	t	Sig.
Constant		1.460	0.148
Muscle %	-0.028	-0.159	0.874
Sedentary time per day (minutes)	0.260	2.354	0.021
Total METs-min	0.184	1.306	0.195
Systolic blood pressure	-0.010	-0.073	0.942
Diastolic blood pressure	0.260	2.498	0.034

## Discussion

Our study aimed to investigate the relationship between HbA1c levels, physical fitness, and anthropometric measurements among young female university students. Through a cross-sectional analysis of 86 participants, we explored various factors potentially influencing glycemic control in this cohort. Initially, we examined anthropometric parameters such as weight, BMI, skin-fold thickness, waist-to-hip ratio, fat percentage, muscle percentage, and visceral fat. Despite a comprehensive assessment, some measurements showed a significant association with HbA1c levels in our study population. This suggests that while these factors are important indicators of overall health and body composition, they may not directly correlate with long-term glycemic control among young females without diabetes.

The mean HbA1c value of 4.69 ± 0.82% observed in our participants falls within the normal range recommended by the American Diabetes Association, indicating good overall glycemic control [3]. Despite the mean BMI of 25.69 ± 4.44 kg/m², which categorizes our participants as overweight according to WHO criteria, it did not identify a significant relationship between BMI and HbA1c levels [11]. This suggests that in this population, BMI alone may not be a sensitive indicator of glucose metabolism or diabetes risk [12-14].

In evaluating physical fitness and activity, we focused on metrics such as total METs-min, days engaged in moderate or vigorous physical activity, and daily sedentary time. Interestingly, we found a positive correlation between sedentary time per day (mean 550.23 ± 302.78 minutes) and HbA1c levels (r = 0.206, 95% CI-0.007 to 0.4). This finding underscores the potential impact of sedentary behavior on glycemic control, highlighting the importance of reducing prolonged sitting periods even in young, apparently healthy individuals [15,16].

Regarding blood pressure measurements, both systolic (SBP) and diastolic blood pressure (DBP) were within normal ranges, with DBP showing a significant correlation with HbA1c levels (r = 0.227, 95% CI 0.016-0.419). The linear regression analysis confirmed that DBP and sedentary hours per day were independently associated with HbA1c levels (p < 0.05), as shown in Table 2, suggesting that both factors contribute to variations in glycemic control among our participants [17].

Our study shares similarities and differences with several previous research studies examining the association between HbA1c levels and various demographic and physiological factors. For instance, Younes et al. conducted a study among Lebanese students aged 18 to 25 years, focusing on BMI, BP, and HbA1c. They found significant correlations between BMI, SBP, DBP, and HbA1c levels, highlighting BMI as a predictor for both HbA1c and BP [18]. In contrast to our findings, they did not observe associations between HbA1c and physical activity levels or sedentary behaviors among their cohort.

In Japan, Takase et al. explored the relationship between fat mass index (FMI), fat-free mass index (FFMI), and HbA1c levels in a large cohort aged ≥ 20 years. They observed that higher FMI and FFMI were associated with elevated HbA1c levels, emphasizing the role of body composition in glycemic control. While our study did not directly measure FMI or FFMI, we similarly found that sedentary hours and mean blood pressure were significant predictors of HbA1c levels, suggesting overlapping concerns regarding lifestyle factors and metabolic health [19].

Furthermore, Hovestadt et al. conducted a population-based cohort study in Germany focusing on children and adolescents, investigating the influence of BMI, age, gender, and pubertal stage on HbA1c levels. They identified positive associations between HbA1c levels and age, gender, and BMI, particularly noting higher HbA1c values among obese participants and those in later pubertal stages [20]. However, our study found that BMI was not significantly associated with HbA1c levels among young female university students, suggesting potential differences in how these factors influence glycemic control in different populations.

Moreover, the study run in Sudan by Omar et al. aimed to investigate the relationship between HbA1c levels and newly diagnosed hypertension in a non-diabetic Sudanese population. Using a cross-sectional design, the researchers assessed HbA1c levels and blood pressure in participants who did not have diabetes but were categorized based on their blood pressure status as either normotensive or hypertensive. The study found that elevated HbA1c levels were significantly associated with higher blood pressure, suggesting that increased HbA1c could be an early indicator of hypertension risk, even in individuals without diabetes [21]. While our study focused on a broader range of factors, including physical activity, BMI, and sedentary behavior, Omar et al. concentrated specifically on the relationship between HbA1c and hypertension. Additionally, our study did not find a significant association between BMI and HbA1c levels, whereas Omar et al.'s research suggests that HbA1c might be a useful indicator for hypertension risk. These results highlight the potential link between glucose metabolism and blood pressure regulation, emphasizing the importance of monitoring HbA1c levels as part of hypertension risk assessment.

Additionally, the study organized by Nakamura et al. explored how physical activity during adolescence affects HbA1c levels in early adulthood. Using a birth cohort design, researchers tracked participants' physical activity levels from adolescence and assessed their HbA1c levels years later. The study found that higher levels of physical activity in adolescence were linked to lower HbA1c levels in early adulthood, suggesting that regular physical activity during teenage years can have long-lasting positive effects on glycemic control [22]. Compared to our study, both studies address the relationship between physical activity and glycemic control. Our study found a correlation between increased sedentary behavior and higher HbA1c levels, indicating the negative impact of reduced physical activity on glycemic control. Similarly, Nakamura et al. highlighted the beneficial effects of higher physical activity on glycemic control, reinforcing the importance of an active lifestyle.

In Korea, Seo et al. investigated the HbA1c distribution among Korean youth and young adults to identify factors influencing high HbA1c levels. Following a search from retrospective studies, they identified parameters such as age, sex, ethnicity, history of parental diabetes, and insulin sensitivity as influential factors in the interpretation of HbA1c levels [23]. Our study, unfortunately, didn’t contain and derive any valuable relationship between HbA1c and the above-mentioned parameters.

In a multilevel analysis carried out in an Iraqi population, Kowsar et al. studied factors influencing HbA1c levels in cohorts of diabetics, non-diabetics, and prediabetics. Factors such as age, sex, BMI, and multiple lipid parameters such as low-density lipoprotein (LDL), high-density lipoprotein (HDL), cholesterol, and triglycerides were taken into account. Out of the above-mentioned factors, BMI tends to be the most significant predictor influencing HbA1c. Unlike our study, BMI and HbA1c showed no correlation and did not take lipid profile into consideration due to the limitations of our study [24].

A study by Kobayashi et al. investigates the relative effectiveness of strength training compared to aerobic exercise and a combined regimen on glycemic control and body composition in normal-weight individuals with type 2 diabetes. This randomized controlled trial (RCT) provides compelling evidence that strength training is more effective than aerobic exercise for managing HbA1c levels among normal-weight individuals with type 2 diabetes. Empowering this demographic with targeted strength training interventions could enhance their glycemic control [25]. However, our study does not consider whether the type of exercise influences the participant’s glycemic control. Also, our study includes healthy subjects with no self-reported status of type 2 diabetes.

The dietary survey done in Japan aimed to delve into the association between macronutrient energy ratios, specifically dietary carbohydrate intake, and HbA1c levels among Japanese patients diagnosed with type 2 diabetes. The primary objective was to identify optimal dietary macronutrient distributions that could enhance glycemic control in the patient population. Multiple regression analysis revealed a significant link between HbA1c levels and carbohydrate intake, controlling for variables such as sex, age, and BMI. The increased carbohydrate intake observed could be reflective of cultural eating patterns and might be contributing to the observed increase in HbA1c levels [26]. The majority of our participants reported eating a mixed diet, and our main aim was to find a correlation between HbA1c, anthropometry, and body composition. Also, our study did not investigate the percentage of carbohydrates and other ratios in their diet while noting the dietary history.

BMI is one of the most commonly used indicators of body fat and overall health due to its simplicity and ease of calculation. It only requires height and weight, making it a beneficial tool in clinical, research, and public health settings. Moreover, its long history of use has established BMI thresholds commonly used to categorize underweight, normal weight, overweight, and obesity, enabling comparisons across studies and populations. However, BMI has many considerable limitations. It does not distinguish between fat and lean body mass, meaning that individuals with high muscle mass may be mistakenly classified as overweight or obese. Similarly, it neglects fat distribution, which is a critical indicator of health risk. BMI cannot account for variations in body shape, sex, age, or ethnicity, which may lead to inaccuracies when assessing cardiometabolic risks or other health conditions. Furthermore, BMI's reliance on a single linear relationship between weight and height does not sufficiently capture the complexity of body composition [27].

The body roundness index (BRI) handles some of BMI's shortcomings by including body shape in its calculation. BRI is derived from height and waist circumference, which allows it to better capture abdominal fat distribution (a key predictor of cardiometabolic risk) [28]. Unlike BMI, BRI provides an estimation of body fat percentage and helps quantify the risk of disorders such as cardiovascular disease and type 2 diabetes. Additionally, BRI is more suggestive of visceral adiposity, which has a more robust correlation with unfavorable health outcomes compared to overall body mass [29].

Recent studies have demonstrated that BRI may surpass BMI in forecasting health risks associated with obesity and metabolic syndrome. Hence, our study explored any relationship between HbA1c and BRI but showed no significance. Its ability to incorporate body roundness and fat distribution makes it a more robust measure for identifying individuals at higher risk, particularly in diverse populations where body shapes vary significantly. Despite these advantages, BRI is less commonly used due to its relative newness and the need for more standardized threshold values across populations. While BRI shows promise, additional research is required to validate its predictive abilities across various demographic groups [30].

This study's strengths lie in its ability to provide a snapshot of a specific cohort at a given point in time, allowing researchers to assess the prevalence of key health indicators and their relationships. By assessing multiple variables simultaneously, this research offers real-time data insights that could kickstart larger, more extensive studies in the future. However, our study also has limitations, including its cross-sectional design, which precludes establishing causality, and restriction to young female participants. The use of convenience sampling and the relatively small sample size may limit the generalizability of our results to broader populations. Additionally, participants' responses regarding their physical activity rely solely on recall and memory, which may affect the accuracy of the results. Our results should be viewed in light of these limitations. Future studies can expand to include dietary status, family history of diabetes, and other comorbid conditions that may influence glycemic control.

## Conclusions

Our research contributes insights into the relationship between sedentary behavior, diastolic blood pressure, and HbA1c levels among a specific cohort of young female university students. While BMI did not emerge as a significant predictor in our study, sedentary hours and diastolic blood pressure were independently associated with HbA1c levels. These findings underscore the importance of considering lifestyle factors beyond traditional anthropometric measurements when assessing diabetes risk and management strategies in young adults. Further research is warranted to explore longitudinal impacts and refine preventive interventions tailored to specific demographic and lifestyle profiles. Hence, the above findings have important implications for diabetic prevention strategies among young women, emphasizing the role of reducing sedentary behavior and maintaining healthy blood pressure levels.

## Appendices

Appendix 1 

**Table 3:** WHO Global Physical Activity Questionnaire (GPAQ)

Physical Activity		
Questions		Response
Activity at work		
1	Does your work involve vigorous-intensity activity that causes large increases in breathing or heart rate, like carrying or lifting heavy loads, digging, or construction work for at least 10 minutes continuously?	Yes: 1; No: 2; If No, go to 4
2	In a typical week, on how many days do you do vigorous-intensity activities as part of your work?	Number of days:
3	How much time do you spend doing vigorous-intensity activities at work on a typical day?	_ _ : _ _ hrs. : mins
4	Does your work involve moderate-intensity activity that causes small increases in breathing or heart rate, such as brisk walking (or carrying light loads) for at least 10 minutes continuously?	Yes: 1; No: 2; If No, go to 7
5	In a typical week, on how many days do you do moderate-intensity activities as part of your work?	Number of days:
6	How much time do you spend doing moderate-intensity activities at work on a typical day?	_ _ : _ _ hrs. : mins
Travel to and from places		
7	Do you walk or use a bicycle (pedal cycle) for at least 10 minutes continuously to get to and from places?	Yes 1;: No: 2; If No, go to 10
8	In a typical week, on how many days do you walk or bicycle for at least 10 minutes continuously to get to and from places?	Number of days --
9	How much time do you spend walking or bicycling for travel on a typical day?	_ _: _ _ hrs. : mins
Recreational activities		
10	Do you do any vigorous-intensity sports, ﬁtness, or recreational (leisure) activities that cause large increases in breathing or heart rate like running or football for at least 10 minutes continuously?	Yes: 1; No: 2; If No, go to 13
11	In a typical week, on how many days do you do vigorous-intensity sports, ﬁtness, or recreational (leisure) activities?	Number of days --
12	How much time do you spend doing vigorous-intensity sports, ﬁtness, or recreational activities on a typical day?	_ _ : _ _ hrs. : mins
Physical activities and recreational activities (cont.)		
13	Do you do any moderate-intensity sports, ﬁtness, or recreational (leisure) activities that cause a small increase in breathing or heart rate, such as brisk walking, (cycling, swimming, volleyball) for at least 10 minutes continuously?	Yes: 1; No: 2; If No, go to 16
14	In a typical week, on how many days do you do moderate-intensity sports, ﬁtness, or recreational (leisure) activities?	Number of days --
15	How much time do you spend doing moderate-intensity sports, ﬁtness, or recreational (leisure) activities on a typical day?	_ _ : _ _ hrs. : mins
Sedentary behaviour		
16	How much time do you usually spend sitting or reclining on a typical day?	_ _ : _ _ hrs.: mins

## References

[1] Sherwani SI, Khan HA, Ekhzaimy A, Masood A, Sakharkar MK (2016). Significance of HbA1c test in diagnosis and prognosis of diabetic patients. Biomark Insights.

[2] Sulaiman N, Mahmoud I, Hussein A, Elbadawi S, Abusnana S, Zimmet P, Shaw J (2018). Diabetes risk score in the United Arab Emirates: a screening tool for the early detection of type 2 diabetes mellitus. BMJ Open Diabetes Res Care.

[3] American Diabetes Association (2011). Diagnosis and classification of diabetes mellitus. Diabetes Care.

[4] Ley SH, Hamdy O, Mohan V, Hu FB (2014). Prevention and management of type 2 diabetes: dietary components and nutritional strategies. Lancet.

[5] Flint A, Arslanian S (2011). Treatment of type 2 diabetes in youth. Diabetes Care.

[6] (2021). World Health Organization (WHO): Global physical activity questionnaire (GPAQ). https://www.who.int/publications/m/item/global-physical-activity-questionnaire.

[7] Zhang R, Dong SY, Wang F, Ma C, Zhao XL, Zeng Q, Fei A (2018). Associations between body composition indices and metabolic disorders in Chinese adults: a cross-sectional observational study. Chin Med J (Engl).

[8] Thomas DM, Bredlau C, Bosy-Westphal A (2013). Relationships between body roundness with body fat and visceral adipose tissue emerging from a new geometrical model. Obesity (Silver Spring).

[9] Gupta S, Jain U, Chauhan N (2017). Laboratory diagnosis of HbA1c: a review. J Nanomed Res.

[10] Park JH, Jo YI, Lee JH (2018). Clinical usefulness of bioimpedance analysis for assessing volume status in patients receiving maintenance dialysis. Korean J Intern Med.

[11] WHO Consultation on Obesity (1999). Obesity: Preventing and Managing the Global Epidemic: Report of a WHO Consultation.

[12] Lin W (2024). The association between body mass index and glycohemoglobin (HbA1c) in the US population’s diabetes status. Int J Environ Res Public Health.

[13] Jo A, Mainous AG 3rd (2018). Informational value of percent body fat with body mass index for the risk of abnormal blood glucose: a nationally representative cross-sectional study. BMJ Open.

[14] Lee DH, Keum N, Hu FB, Orav EJ, Rimm EB, Willett WC, Giovannucci EL (2018). Comparison of the association of predicted fat mass, body mass index, and other obesity indicators with type 2 diabetes risk: two large prospective studies in US men and women. Eur J Epidemiol.

[15] Al-Goblan AS, Al-Alfi MA, Khan MZ (2014). Mechanism linking diabetes mellitus and obesity. Diabetes Metab Syndr Obes.

[16] Chan JC, Malik V, Jia W, Kadowaki T, Yajnik CS, Yoon KH, Hu FB (2009). Diabetes in Asia: epidemiology, risk factors, and pathophysiology. JAMA.

[17] Mills KT, Bundy JD, Kelly TN (2016). Global disparities of hypertension prevalence and control: a systematic analysis of population-based studies from 90 countries. Circulation.

[18] Younes N, Atallah M, Alam R, Chehade NH, Gannagé-Yared MH (2019). HbA1c and blood pressure measurements: relation with gender, body mass index, study field, and lifestyle in Lebanese students. Endocr Pract.

[19] Takase M, Nakamura T, Hirata T (2022). Association between fat mass index, fat-free mass index and hemoglobin A1c in a Japanese population: the Tohoku Medical Megabank Community-based Cohort Study. J Diabetes Investig.

[20] Hovestadt I, Kiess W, Lewien C (2022). HbA1c percentiles and the association between BMI, age, gender, puberty, and HbA1c levels in healthy German children and adolescents. Pediatr Diabetes.

[21] Omar SM, Musa IR, Abdelbagi O, Sharif ME, Adam I (2022). The association between glycosylated haemoglobin and newly diagnosed hypertension in a non-diabetic Sudanese population: a cross-sectional study. BMC Cardiovasc Disord.

[22] Nakamura PM, Mielke GI, Horta BL (2017). Physical activity throughout adolescence and HbA1c in early adulthood: birth cohort study. J Phys Act Health.

[23] Seo JY, Hwang SS, Kim JH, Lee YA, Lee SY, Shin CH, Yang SW (2018). Distribution of glycated haemoglobin and its determinants in Korean youth and young adults: a nationwide population-based study. Sci Rep.

[24] Kowsar R, Mansouri A (2022). Multi-level analysis reveals the association between diabetes, body mass index, and HbA1c in an Iraqi population. Sci Rep.

[25] Kobayashi Y, Long J, Dan S (2023). Strength training is more effective than aerobic exercise for improving glycaemic control and body composition in people with normal-weight type 2 diabetes: a randomised controlled trial. Diabetologia.

[26] Yamakawa T, Sakamoto R, Takahashi K (2019). Dietary survey in Japanese patients with type 2 diabetes and the influence of dietary carbohydrate on glycated hemoglobin: the Sleep and Food Registry in Kanagawa study. J Diabetes Investig.

[27] National Academies of Sciences, Engineering Engineering, and Medicine (2023). Translating Knowledge of Foundational Drivers of Obesity into Practice: Proceedings of a Workshop Series. Foundational Drivers of Obesity into Practice: Proceedings of a Workshop Series [Internet]. National Academies Press (US.

[28] Zhang X, Ma N, Lin Q (2024). Body roundness index and all-cause mortality among US adults. JAMA Netw Open.

[29] Chang Y, Guo X, Chen Y (2015). A body shape index and body roundness index: two new body indices to identify diabetes mellitus among rural populations in northeast China. BMC Public Health.

[30] Calderón-García JF, Roncero-Martín R, Rico-Martín S (2021). Effectiveness of body roundness index (BRI) and a body shape index (ABSI) in predicting hypertension: a systematic review and meta-analysis of observational studies. Int J Environ Res Public Health.

